# Lyophilized allogeneic bone tissue as an antibiotic carrier

**DOI:** 10.1007/s10561-016-9582-5

**Published:** 2016-09-08

**Authors:** Débora C. Coraça-Huber, Christoph G. Ammann, Michael Nogler, Manfred Fille, Lars Frommelt, Klaus-Dieter Kühn, Christian Fölsch

**Affiliations:** 1 Experimental Orthopaedics, Department of Orthopaedic Surgery, Medical University of Innsbruck, Innrain 36, 6020 Innsbruck, Austria; 2 Division of Hygiene and Medical Microbiology, Medical University Innsbruck, Schöpfstr. 41, 3rd Floor, Innsbruck, Austria; 3 Institute for Infectiology, Clinical Microbiology and Hospital Care, ENDO Clinic Hamburg, Holstenstraße 2, Hamburg, Germany; 4 Department of Orthopaedics and Orthopaedic Surgery, Medical University Graz, Auenbruggerplatz 5, Graz, Austria; 5 Department of Orthopaedics and Orthopaedic Surgery, Medical University of Giessen, Baldingerstraße, Marburg, Germany

**Keywords:** Bone grafts, Antibiotics, Lyophilized bone chips, Local delivery, Joint infection

## Abstract

The rising number of primary joint replacements worldwide causes an increase of revision surgery of endoprostheses due bacterial infection. Revision surgery using non-cemented implants seems beneficial for the long-term outcome and the use of antibiotic-impregnated bone grafts might control the infection and give a good support for the implant. In this study we evaluated the release of antibiotics from fresh-frozen and lyophilized allogeneic bone grafts. Lyophilized bone chips and fresh frozen bone chips were mixed with gentamicin sulphate, gentamicin palmitate, vancomycin, calcium carbonate/calcium sulphate impregnated with gentamicin sulphate, and calcium carbonate/calcium sulphate bone substitute material impregnated with vancomycin. The efficacy of each preparation was measured by drug release tests and bacterial susceptibility using *B. subtilis*, *S. aureus* and methicillin-resistant *Staphylococcus aureus*. The release of gentamicin from lyophilized bone was similar to the release rate from fresh frozen bone during all the experimental time. That fact might be related to the similar porosity and microstructure of the bone chips. The release of gentamicin from lyophilized and fresh frozen bone was high in the first and second day, decreasing and keeping a low rate until the end of the second week. Depending on the surgical strategy either polymethylmethacrylate or allogeneic bone are able to deliver sufficient concentrations of gentamicin to achieve bacterial inhibition within two weeks after surgery. In case of uncemented revision of joint replacements allogeneic bone is able to deliver therapeutic doses of gentamicin and peak levels immediately after implantation during a fortnight. The use of lyophilized and fresh frozen bone allografts as antibiotic carriers is recommended for prophylaxis of bone infection.

## Introduction

Bone grafts are used for reconstructing bone defects caused by implant associated complications, trauma and tumors (Putzer et al. 2011; Hinsenkamp et al. 2012). While autografts can be used, donor site morbidity can be avoided using allografts. (Barbour and King 2003; Butler et al. 2005; Haimi et al. 2008). Bone grafts might derive from post mortem donors or might be donated from femoral heads of living patients undergoing hip arthroplasty creating bone chips to fill bone defects during revision surgery of joint replacements since impaction bone grafting increases primary stability and bone stock which is essential for the longevity of the implant.

However, fresh frozen bone chips bear a higher risk of transmission of diseases and local contamination compared with processed bone grafts (Brewster et al. 1999; Hofmann et al. 2005). Surgery with bone allografts is complex and time-consuming; therefore it is per se prone to a higher infection rate (2.0–2.5 %) (Blom et al. 2003; Parvizi et al. 2007, 2008). Additionally, the impaction used for placing bone transplants can disrupts the local circulation and reduce the bone ingrowth (Tagil and Aspenberg 1998; Duffy et al. 2007; Buttaro et al. 2012; Ding et al. 2015). In the case of a site infection, systemically administered antibiotics cannot reach the infected bone graft (Isefuku et al. 2003). As a known complication factor, biofilms can be formed on the surface of foreign materials thus increasing antibiotic resistance (Coraça-Huber et al. 2012a, b). Staphylococcus epidermidis and *Staphylococcus aureus* are the germs which mostly colonize implant surfaces (Christensen et al. 1989).

The number of infection related to multi resistant bacteria is increasing (Ascherl 2010). Also, biofilm forming bacteria is a major concern for treatment of implant-related infections (Costerton et al. 1999; Patel 2005; Frommelt 2006; Esteban et al. 2010; Coraça-Huber et al. 2012a). Biofilm has been defined as multicellular community composed of prokaryotic and or eukaryotic cells embedded in matrix (Frommelt 2004; Esteban et al. 2010). In this case, sessile bacteria become antibiotic-resistant making treatment and diagnosis difficult (Patel 2005). Antibiotic treatment are directed against planktonic bacteria which relieves symptoms but does not cure the infection and therefore might delay adequate treatment (Frommelt 2006). High antibiotic concentrations at the implantation site, immediately available after surgery, should prevent development of biofilm.

Antibiotics delivered from an implanted biomaterial can be potentially used to prevent infections caused by biofilm formation, providing high concentrations of antibiotics at the surgical site without local or systemic toxicity. In addition, these materials should be osteoconductive and osteoinductive, thus supporting bone healing without further surgery (Saraf et al. 2010). Promising results have been achieved using bone substitutes or bone grafts mixed with bone substitutes and antibiotics. Among a broad variety of materials, calcium sulphate and calcium carbonate beads proved to be a suitable osteoconductive material for bone reconstruction (Wichelhaus et al. 2001; Evaniew et al. 2013; Roberts et al. 2013; Coraça-Huber et al. 2015).

Gentamicin sulphate (GS) salt is commonly used antibiotic for local application in orthopaedic surgery, for example mixed with PMMA cements. Gentamicin base (GB) consists of a mixture of gentamicin C1, C1a and C2 a + b. Gentamicin sulphate is highly water soluble. This substance can be used as a coating material for biomaterials and tissues by turning the water-soluble GS into a low-soluble gentamicin fatty acid salt (converting gentamicin sulphate to gentamicin palmitate; GP) (Kühn et al. 2003; Kuhn et al. 2008; Coraça-Huber et al. 2013a, b). Herafill^®^ powder is used in the composition of bioabsorbable beads and is composed of calcium sulphate, calcium carbonate and glycerine tripalmitate as bonding additive. It contains 1 % of GS corresponding to 2.5 g of GB. Herafill^®^ is also manufactured as granules to be used as a bone void filling material as well as an antibiotic carrier (Coraça-Huber et al. 2014).

In this study we evaluated two different preparations of femoral heads allografts as antibiotic carrier. Lyophilized bone chips and fresh frozen bone chips were mixed with gentamicin sulphate, gentamicin palmitate, vancomycin, calcium carbonate/calcium sulphate impregnated with gentamicin sulphate, and calcium carbonate/calcium sulphate bone substitute material impregnated with vancomycin. The efficacy of each preparation was measured by drug release tests and bacterial susceptibility using *B. subtilis*, *S. aureus* and methicillin-resistant *Staphylococcus aureus*.

## Materials and methods

### Bone tissue

Different preparation of femoral heads allografts obtained from living donors (telos GmbH, Marburg, Germany and Tissue Bank, Charité–Medical University Berlin, Berlin, Germany) were used as antibiotic carrier. Two different preparations were tested: lyophilized bone chips (BChT) and fresh frozen bone chips (FF-BChT). All patients gave their written consent that the removed tissue was allowed to be used for research purposes.

### Antibiotic and reference substances

Gentamicin sulphate (GS), gentamicin palmitate (GP), vancomycin (V), calcium carbonate/calcium sulphate bone substitute material impregnated with 5 and 10 % gentamicin sulphate (HeraG; Herafill^®^) and calcium carbonate/calcium sulphate bone substitute material impregnated with 5 and 10 % vancomycin hydrochloride (HeraV; Herafill^®^) were used in this study. Polymethylmethacrylate beads impregnated with gentamicin sulfate (Heraeus PMMA Chain G30, Heraeus Medical GmbH, Wehrheim, Germany; approximately 4.5 mg gentamicin per bead) and calcium carbonate/calcium sulfate bone substitute beads impregnated with gentamicin sulfate (Herafill^®^ beads G^®^, Heraeus Medical GmbH, Wehrheim, Germany; 2.5 mg gentamicin per bead) were used as reference materials. Also, FF-BChT (telos GmbH, Marburg, Germany and Tissue Bank, Charité–Medical University Berlin, Berlin, Germany) samples were used as reference materials.

### Microorganisms

*Bacillus subtilis* (Merck KGaA, Germany in Test Agar pH 8.0 Merck KGaA, Germany), *Staphylococcus aureus* ATCC 29213 (American Type Culture Collection, LGC Standards GmbH, Wesel, Germany) and methicillin-resistant *Staphylococcus aureus* MRSA DSM 46320 (Leibniz Institute DSMZ Deutsche Sammlung von Mikroorganismen und Zellkulturen— German Collection of Microorganisms and Cell Cultures, Braunschweig, Germany) were used for antibiotic delivery and antibiotic susceptibility assays.

### Gentamicin base release

To evaluate the release rate of antibiotics from allografts, the BChT samples were mixed with GS, GS + GP, V, HeraG, HeraV. The exactly concentration of each mixtures is detailed on Table 1. The antibiotic release assay was carried out using phosphate-buffered saline (PBS) pH 7.4 (Sigma-Aldrich, Schnelldorf, Germany). For that, 3 ml of PBS were added into each tube containing 1 cm^3^ of each BChT mixture. The tubes were vortexed for 1 min and placed on a shaker at 37 °C. Daily, from 1 to 14 days, the elution medium was completely removed and fresh PBS was added. The removed elution was vortexed and stored at −20 °C until the tests.

**Table 1:** Mixtures used for antibiotic delivery tests

Mixtures used for the antibiotic delivery assay	
Test groups	Concentration mixtures
BChT + GS/GP (1.6 % GB)	1 g BChT + 0.016 g GB (0.014 g GS) + 0.016 g GB (0.04 g GP)
BChT + GS/GP (3.2 % GB)	1 g BChT + 0.032 g GB (0.028 g GS) + 0.032 g GB (0.08 g GP)
BChT + GS/GP (6.4 % GB)	1 g BChT + 0.064 g GB (0.056 g GS) + 0.064 g GB (0.16 g GP)
BChT + HeraG 10 % (1.6 %GB)	1 g BChT + 0.016 GB (0.16 g GS)
BChT + HeraG 10 % (3.2 %GB)	1 g BChT + 0.032 GB (0.32 g GS)
BChT + HeraG 10 % (6.4 %GB)	1 g BChT + 0.064 GB (0.64 g GS)
BChT + HeraG 5 % (6.4 %GB)	1 g BChT + 0.064 (1.28 g GS)
BChT + HeraV 10 % (2 %VB)	1 g BChT + 0.02 g VB (0.2 g V)
BChT + HeraV 10 % (4 %VB)	1 g BChT + 0.04 g VB (0.4 g V)
BCht + HeraV 10 % (8 %VB)	1 g BChT + 0.08 g VB (0.8 g V)
BCht + HeraV 5 % (8 %VB)	1 g BChT + 0.08 g VB (1.6 g V)

Reference groups	Concentration mixtures
BChT + GS	1 g BChT + 1 mL (gentamicin 1 mg/mL) (Witso et al. 2005)
BChT + V	1 g BChT + 1 mL (vancomycin 1 mg/mL) (Witso et al. 2005)
PMMA beads	≃1 g gentamicin sulfate/bead
Herafill beads	2.5 mg gentamicin sulfate/bead
FF-BChT + GS + GP (1.6 % GB)	1 g FF-BChT + 0.016 g GB (0.014 g GS) + 0.016 g GB (0.04 g GP)
FF-BChT + GS/GP (3.2 % GB)	1 g FF-BChT + 0.032 g GB(0.028 g GS) + 0.032 g GB (0.08 g GP)
FF-BChT + GS/GP + (6.4 % GB)	1gFF-BChT + 0.064 g GB(0.056 g GS) + 0.064 g GB (0.16 g GP)

Test groups: lyophilized bone chips mixed with gentamicin sulfate and gentamicin palmitate powder (BChT + GS/GP); gentamicin base powder (GB); lyophilized bone chips mixed with calcium carbonate/calcium sulphate impregnated with gentamicin sulphate powder (BChT + HeraG); lyophilized bone chips mixed with calcium carbonate/calcium sulphate granulate impregnated with vancomycin (BChT + HeraV); Reference groups: lyophilized bone chips impregnated with gentamicin sulphate by immersion (BChT + GS) (Witso et al. 2005); lyophilized bone chips impregnated with vancomycin by immersion (BChT + V) (Witso et al. 2005) polymethylmethacrylate beads impregnated with gentamicin sulfate (PMMA beads); calcium carbonate/calcium sulfate bone substitute beads impregnated with gentamicin sulfate (Herafill beads); fresh frozen bone chips mixed with gentamicin sulfate and gentamicin palmitate (FF-BChT + GS/GP). The mixtures were kept for 24 h prior de addition of PBS for the elution tests

### *Bacillus subtilis* assay for estimation of antibiotic release concentrations

Concentrations of the delivered antibiotic in the elution were determined by a conventional microbiological agar diffusion assay using *Bacillus subtilis* as the indicator strain already described by Coraça-Huber et al. (2015). Using a 6-mm diameter metal punch, a hole was made at the center of each *B. subtilis* agar plate into which 100 μL of each collected elution or 100 μL of 10-fold dilutions of each standard concentration was added. The plates containing the samples were incubated at 37 °C for 24 h. After the incubation, the diameter of the zones of inhibition in centimeters (cm) was measured for each plate with a ruler. The diameter was confirmed with a second measurement. The size of punched area was subtracted for the final measurement. The standard curve was obtained by logarithmic regression and used to predict the concentration of GB in each elution. This assay was carried out in triplicate.

### *Staphylococcus aureus* and methicillin-resistant *Staphylococcus aureus* susceptibility tests

*Staphylococcus aureus* ATCC 29213 and methicillin-resistant *Staphylococcus aureus* MRSA DSM 46320 suspensions at 2 × 10^5^ cells (0.5 McFarland) were prepared and 10 μL were inoculated using Müller-Hinton agar plates. With a 6-mm diameter metal punch, a hole was made on the center of each plate where 100 μL of each sample was added. The plates were incubated at 37 °C for 24 h. After 24 h, the zones of inhibition were measured on each plate with a ruler. The diameter was confirmed with a second measurement. The size of the punched area was subtracted for the final measurement. These tests were carried out in triplicate.

### Statistical analysis

Statistical evaluation was carried out to detect differences between the delivery rate and susceptibility tests between the samples tested. To evaluate the differences between the samples taking into consideration the elution concentration along the time Two-way ANOVA with Bonferroni’s multiple comparisons test was applied. To detect the cumulative differences and susceptibility of microorganisms between the mixtures One-way ANOVA with Bonferroni’s multiple comparisons test was carried out.

## Results

### *Bacillus subtilis* assay for estimation of antibiotic release concentrations

**Fig. 1:**
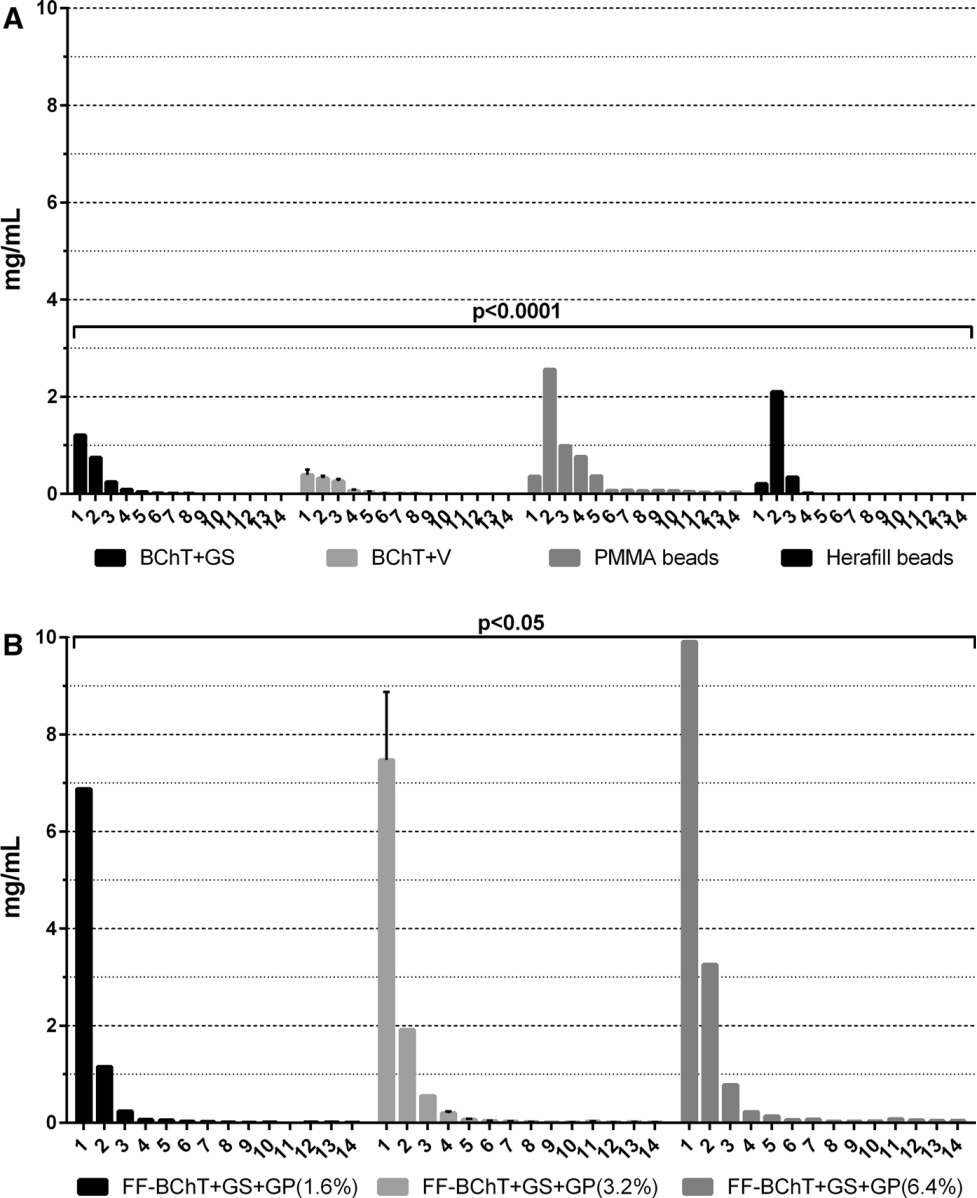
Reference groups: antibiotic release concentrations obtained from *Bacillus subtilis* assay. **a** Lyophilized bone chips impregnated with gentamicin sulphate by immersion (1 mg/mL; BChT + GS); lyophilized bone chips impregnated with vancomycin sulphate by immersion (1 mg/mL; BChT + V) polymethylmethacrylate beads impregnated with gentamicin sulfate (PMMA beads); calcium carbonate/calcium sulfate bone substitute beads impregnated with gentamicin sulfate (Herafill beads); **b** fresh-frozen bone fragments mixed with gentamicin sulfate and gentamicin palmitate 1.6 % of gentamicin base (FF-BChT + GS + GP 1.6 %); fresh-frozen bone fragments mixed with gentamicin sulfate and gentamicin palmitate 3.2 % of gentamicin base (FF-BChT + GS + GP 3.2 %); fresh-frozen bone fragments mixed with gentamicin sulfate and gentamicin palmitate 6.4 % of gentamicin base (FF + BChT + GS + GP 6.4 %)

**Fig. 2:**
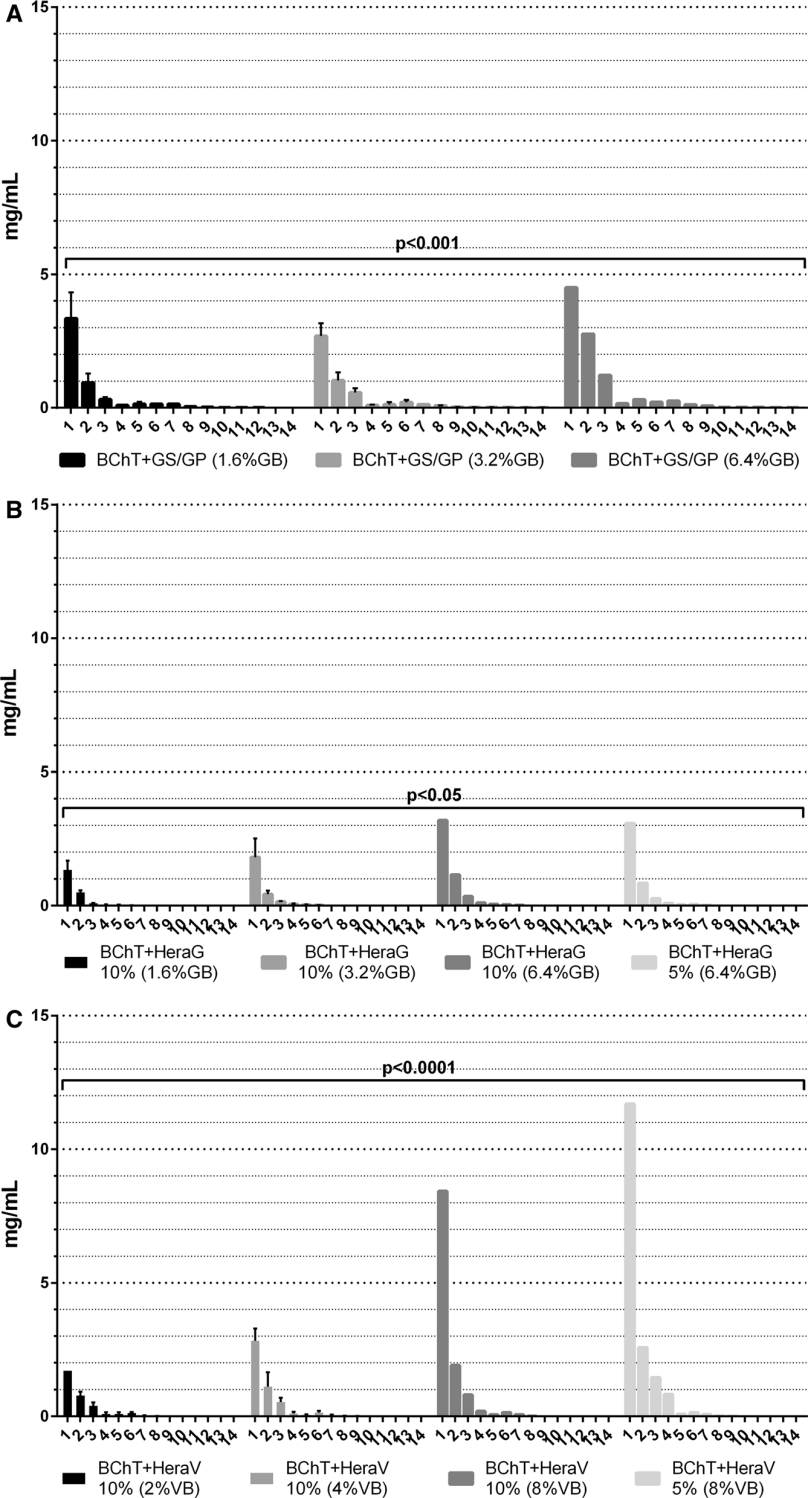
Test groups: antibiotic release concentrations obtained from *Bacillus subtilis* assay. **a** Lyophilized bone chips mixed with gentamicin sulfate and gentamicin palmitate 1.6 % of gentamicin base (BChT + GS + GP 1.6 %); lyophilized bone chips mixed with gentamicin sulfate and gentamicin palmitate 3.2 % of gentamicin base (BChT + GS + GP 3.2 %); lyophilized bone chips mixed with gentamicin sulfate and gentamicin palmitate 6.4 % of gentamicin base (BChT + GS + GP 6.4 %); **b** lyophilized bone chips mixed with HerafillG 10 % with 1.6 % gentamicin base (BChT + HeraG 10 %—1.6 %GB); lyophilized bone chips mixed with HerafillG 10 % with 3.2 % gentamicin base (BChT + HeraG 10 %—3.2 %GB); lyophilized bone chips mixed with HerafillG 10 % with 6.4 % gentamicin base (BChT + HeraG 10 %—6.4 %GB); lyophilized bone chips mixed with HerafillG 5 % with 6.4 % gentamicin base (BChT + HeraG 5 %—6.4 %GB); c lyophilized bone chips mixed with HerafillV 10 % with 2 % vancomycin base (BChT + HeraV 10 %—2 %GB); lyophilized bone chips mixed with Herafill V 10 % with 4 % vancomycin base (BChT + HeraV 10  %—4 %GB); lyophilized bone chips mixed with HerafillV 10 % with 8 % vancomycin base (BChT + HeraV 10 %—8 %GB); lyophilized bone chips mixed with HerafillV 10 % with 8 % vancomycin base (BChT + HeraV 5 %—8 %GB)

Taking into consideration the release of antibiotics from the BChT and PMMA and Herafill beads we could observe that the BChT + GS and PMMA beads showed a significant higher delivery rate in comparison with BChT + V and Herafill beads. PMMA beads were the only material that allowed delivery of antibiotic at least until the 14th day. However, the significant release was observed only until the 5th day (*p* < 0.0001; Fig. 1a). The antibiotic delivery rate from fresh frozen samples followed the concentration of GS + GP from each group where FF-BChT + GS + GP 6.4 % showed significant higher concentration in comparison with FF-BChT + GS + GP 1.6 and 3.2 %. The highest concentration were detected until the 5th day of elution (*p* < 0.05; Fig. 1b). The antibiotic concentration in the elution of BChT + GS + GP also followed the mixture concentration. Here BChT + GS + GP 6.4 % showed higher antibiotic release when compared to BChT + GS + GP 1.6 and 3.2 %. The highest delivery was detected only until the 3rd elution day (*p* < 0.001; Fig. 2a). The antibiotic delivery from the BChT mixed with HeraG also followed the concentration of the mixtures made prior the elution tests. Here BChT + HeraG 10 % (6.4 % GB) and BChT + HeraG 5 % (6.4 %GB) showed no significant differences between the concentrations of antibiotic released. The two groups showed the highest levels of antibiotic being released until the 4th elution day. Although, BChT + HeraG 10 % (6.4 %GB) and BChT + HeraG 5 % (6.4 %GB) delivery significant different concentration (*p* < 0.05) of the antibiotic in comparison to BChT + HeraG 10 % (1.6 %GB) and BChT + HeraG 10 % (3.2 %GB). In these two groups the highest delivery was detected until the 2nd elution day (Fig. 2b). A similar pathway could be observed between the groups of BChT mixed with HeraV. Here BChT + HeraV (8 %VB) and BChT + HeraV 5 % (8 % VB) showed very high concentration release in comparison with BChT + HeraV 10 % (2 % VB) and BChT + HeraV 10 % (4 % VB). However BChT + HeraV 10 % (8 % VB) showed significant higher delivery (*p* < 0.05) in comparison with BChT + HeraV 10 % (2 % VB) and in comparison with BChT + HeraV 10 % (2 % VB) and BChT + HeraV 10 % (4 % VB; *p* < 0.0001). Also here, the highest delivery rate was detected until the 3rd and 4th elution day (Fig. 2c).

### *Staphylococcus aureus* and methicillin-resistant *Staphylococcus aureus* susceptibility tests

**Fig. 3:**
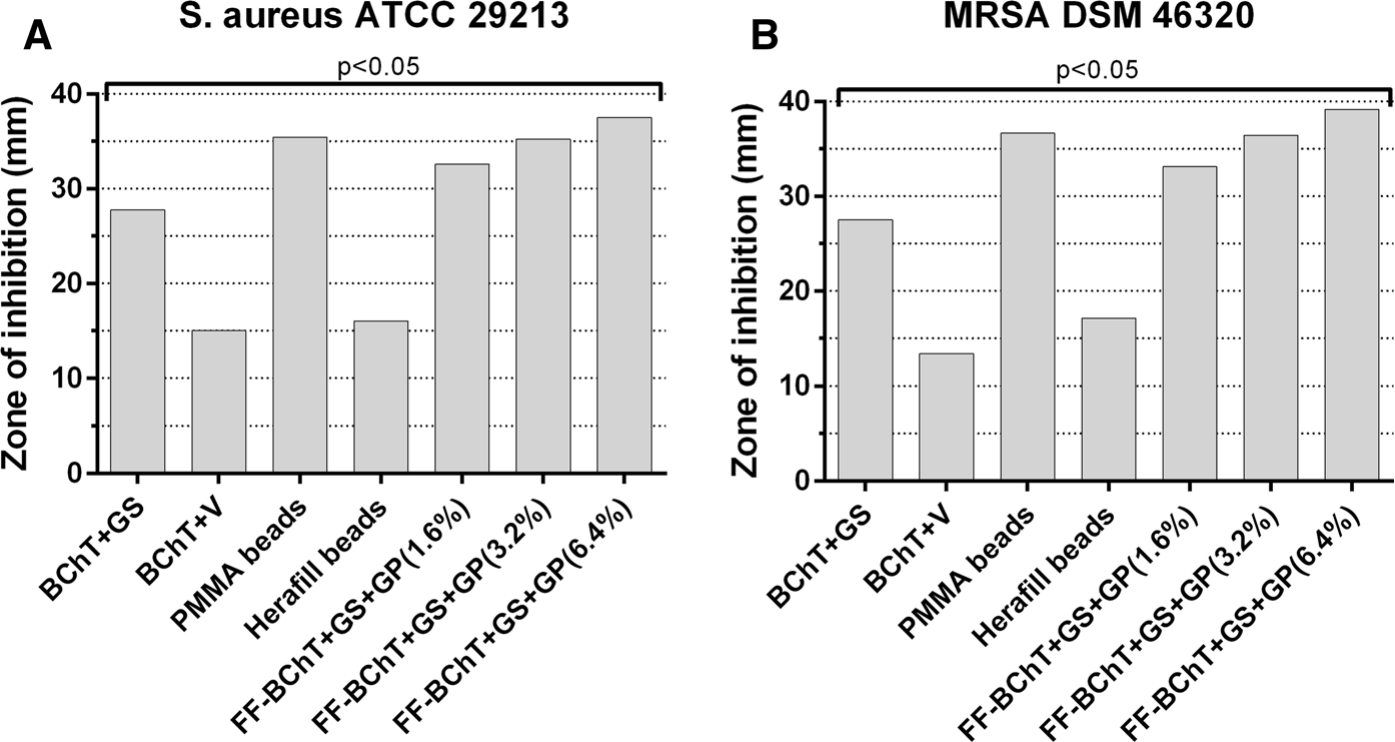
Cumulative results for susceptibility tests of antibiotic released against **a** *S. aureus* ATCC 29213 and **b** MRSA DSM 46320. Reference groups: Lyophilized bone chips impregnated with gentamicin sulphate by immersion (1 mg/mL; BChT + GS); lyophilized bone chips impregnated with vancomycin sulphate by immersion (1 mg/mL; BChT + V) polymethylmethacrylate beads impregnated with gentamicin sulfate (PMMA beads); calcium carbonate/calcium sulfate bone substitute beads impregnated with gentamicin sulfate (Herafill beads); fresh-frozen bone fragments mixed with gentamicin sulfate and gentamicin palmitate 1.6 % of gentamicin base (FF-BChT + GS + GP 1.6 %); fresh-frozen bone fragments mixed with gentamicin sulfate and gentamicin palmitate 3.2 % of gentamicin base (FF-BChT + GS + GP 3.2 %); fresh-frozen bone fragments mixed with gentamicin sulfate and gentamicin palmitate 6.4 % of gentamicin base (FF + BChT + GS + GP 6.4 %)

**Fig. 4:**
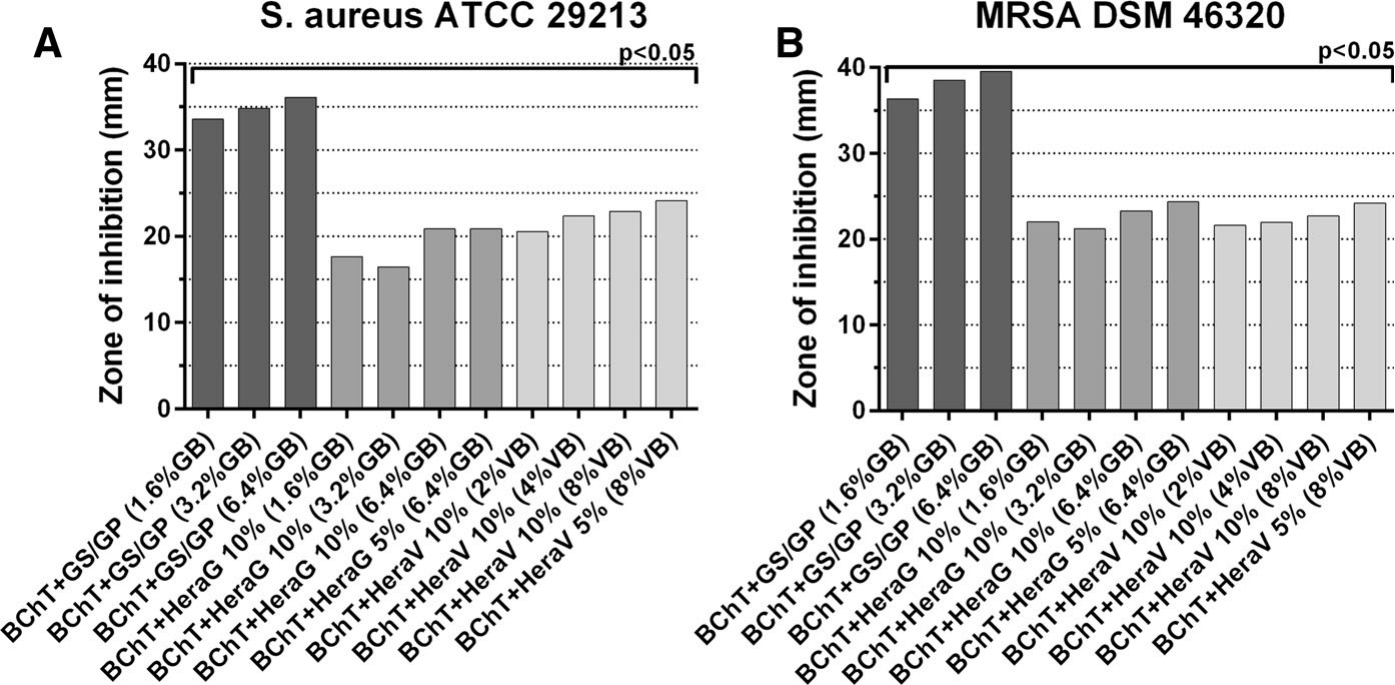
Cumulative results for susceptibility tests of antibiotic released against **a** *S. aureus* ATCC 29213 and **b** MRSA DSM 46320. Test groups: lyophilized bone chips mixed with gentamicin sulfate and gentamicin palmitate 1.6 % of gentamicin base (BChT + GS + GP 1.6 %); lyophilized bone chips mixed with gentamicin sulfate and gentamicin palmitate 3.2 % of gentamicin base (BChT + GS + GP 3.2 %); lyophilized bone chips mixed with gentamicin sulfate and gentamicin palmitate 6.4 % of gentamicin base (BChT + GS + GP 6.4 %); lyophilized bone chips mixed with HerafillG 10 % with 1.6 % gentamicin base (BChT + HeraG 10 %—1.6 %GB); lyophilized bone chips mixed with HerafillG 10 % with 3.2 % gentamicin base (BChT + HeraG 10 %—3.2 %GB); lyophilized bone chips mixed with HerafillG 10 % with 6.4 % gentamicin base (BChT + HeraG 10 %—6.4 %GB); lyophilized bone chips mixed with HerafillG 5 % with 6.4 % gentamicin base (BChT + HeraG 5 %—6.4 %GB); lyophilized bone chips mixed with HerafillV 10 % with 2 % vancomycin base (BChT + HeraV 10 %—2 %GB); lyophilized bone chips mixed with Herafill V 10 % with 4 % vancomycin base (BChT + HeraV10 %—4 %GB); lyophilized bone chips mixed with HerafillV 10 % with 8 % vancomycin base (BChT + HeraV 10 %—8 %GB); lyophilized bone chips mixed with HerafillV 10 % with 8 % vancomycin base (BChT + HeraV 5 %—8 %GB)

**Fig. 5:**
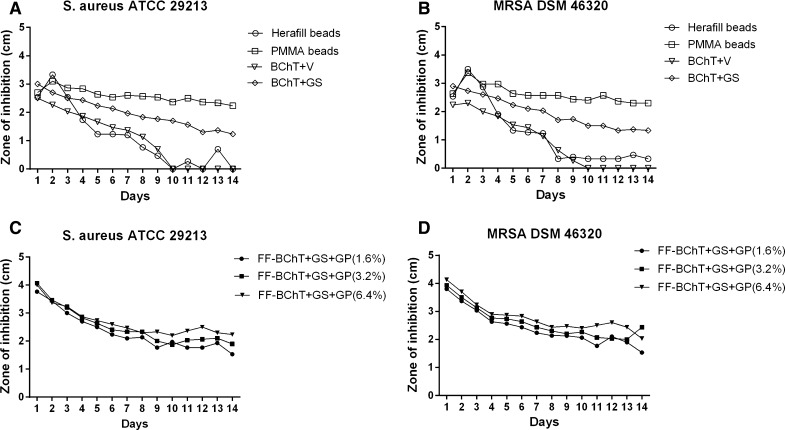
Susceptibility tests of antibiotic released against **a** *S. aureus* ATCC 29213 and **b** MRSA DSM 46320. Reference groups: **a**, **b** lyophilized bone chips impregnated with gentamicin sulphate by immersion (1 mg/mL; BChT + GS); lyophilized bone chips impregnated with vancomycin sulphate by immersion (1 mg/mL; BChT + V) polymethylmethacrylate beads impregnated with gentamicin sulfate (PMMA beads); calcium carbonate/calcium sulfate bone substitute beads impregnated with gentamicin sulfate (Herafill beads); **c**, **d** fresh-frozen bone fragments mixed with gentamicin sulfate and gentamicin palmitate 1.6 % of gentamicin base (FF-BChT + GS + GP 1.6 %); fresh-frozen bone fragments mixed with gentamicin sulfate and gentamicin palmitate 3.2 % of gentamicin base (FF-BChT + GS + GP 3.2 %); fresh-frozen bone fragments mixed with gentamicin sulfate and gentamicin palmitate 6.4 % of gentamicin base (FF + BChT + GS + GP 6.4 %)

**Fig. 6:**
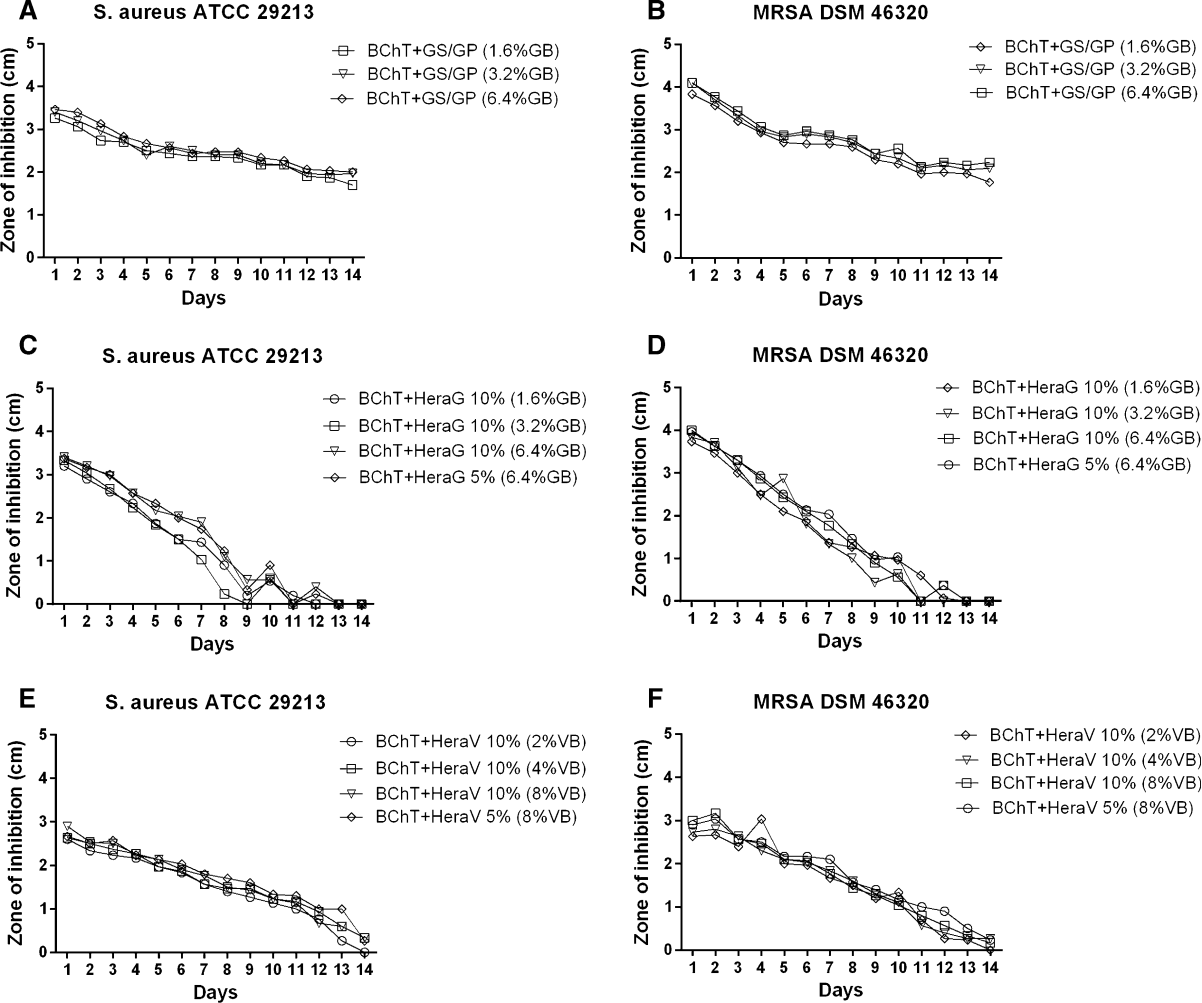
Susceptibility tests of antibiotic released against (**a**, **c**, **e**) *S. aureus* ATCC 29213 and (**b**, **d**, **f**) MRSA DSM 46320. Test groups: **a**, **b** lyophilized bone chips mixed with gentamicin sulfate and gentamicin palmitate 1.6 % of gentamicin base (BChT + GS + GP 1.6 %); lyophilized bone chips mixed with gentamicin sulfate and gentamicin palmitate 3.2 % of gentamicin base (BChT + GS + GP 3.2 %); lyophilized bone chips mixed with gentamicin sulfate and gentamicin palmitate 6.4 % of gentamicin base (BChT + GS + GP 6.4 %); **c**, **d** lyophilized bone chips mixed with HerafillG 10 % with 1.6 % gentamicin base (BChT + HeraG 10 %—1.6 %GB); lyophilized bone chips mixed with HerafillG 10 % with 3.2 % gentamicin base (BChT + HeraG 10 %—3.2 %GB); lyophilized bone chips mixed with HerafillG 10 % with 6.4 % gentamicin base (BChT + HeraG 10 %—6.4 %GB); lyophilized bone chips mixed with HerafillG 5 % with 6.4 % gentamicin base (BChT + HeraG 5 %—6.4 %GB); **e**, **f** lyophilized bone chips mixed with HerafillV 10 % with 2 % vancomycin base (BChT + HeraV 10 %—2 %GB); lyophilized bone chips mixed with Herafill V 10 % with 4 % vancomycin base (BChT + HeraV10 %—4 %GB); lyophilized bone chips mixed with HerafillV 10 % with 8 % vancomycin base (BChT + HeraV 10 %—8 %GB); lyophilized bone chips mixed with HerafillV 10 % with 8 % vancomycin base (BChT + HeraV 5 %—8 %GB)

Here we calculated the cumulative values from all time intervals to show the mixture with better antibacterial effect. Within the group of reference samples, against *S. aureus*, the best activity was observed by PMMA beads and all the FF-BChT + GS + GP. Also BChT + GS showed high efficacy. These mixtures showed significant higher activity (*p* < 0.05) in comparison with the other mixtures. BChT + V and Herafill beads showed less activity and no significant difference between each other (Fig. 3a). Similar results were obtained with the samples tested against MRSA (Fig. 3b). The cumulative results for the susceptibility tests against *S. aureus* and MRSA for the BChT mixed with GS + GP, HeraG and HeraV, showed that the activity of BChT mixed with GS + GP was significantly higher in comparison with BChT mixed with HeraG and HeraV (*p* < 0.05; Fig. 4a, b). Besides the cumulative results for the susceptibility tests against *S. aureus* and MRSA, the measurement of the zone of inhibition is also presented for each time interval. In the reference groups we can also observe the highest efficacy of PMMA beads and BChT + GS against *S. aureus* and MRSA in comparison with BChT + V and Herafill beads (Fig. 5a, b). For the fresh frozen samples, besides the difference in antibiotic concentration, the three groups (FF + BChT + GS + GP 1.6 %, FF + BChT + GS + GP 3.2 %, FF + BChT + GS + GP 6.4 %) showed similar effect against the microorganisms showing its activity until the 14th elution day (Fig. 5c, d). For the samples of the test group, the mixture that most efficiently delivered the antibiotic substances until the last day was BChT + GS + GP, for all concentration of gentamicin base used. Here we can see that even at the last day, all the concentration reached a zone of inhibition of approximately 2 cm. The efficiency of BChT + GS + GP was similar against *S. aureus* and MRSA. The mixtures of BChT with HeraG showed high antimicrobial activity until the end of the first week for *S. aureus* and MRSA. The mixture of BChT with HeraV showed less activity against the microorganisms in comparison with BChT + GS + GP, however, this activity could be observed until the 10th elution day (Fig. 6).

## Discussion

Once an infection is established, the removal of implanted devices is necessary for a proper treatment (Zimmerli et al. 2004; Frommelt 2006). Biomaterial-mediated infections are resistant to antibiotic treatment even at high doses (Gristina 1987). Only early infections might be managed with systemic antibiotic therapy (Zimmerli et al. 2004; Frommelt 2006). Surgical debridement removing all suspicious tissue is essential to achieve control of infection and good long-term results (Frommelt 2006). Early detection of infection and aggressive treatment has a high eradication rate since only 64 % of all infections occurred within the first 12 months of primary surgery (Phillips et al. 2006).

In this study we evaluated two different preparations of femoral heads allografts as antibiotic carrier. Lyophilized and fresh frozen bone chips. Lyophilisation of bone allografts can be conducted under complete screening of donors, does not use any chemical agents for preparation and can help decrease the contaminants. The lyophilisation process of bone causes only a small reduction of pull-out force which is not relevant regarding impaction of bone allograft in revision surgery arthroplasty (Folsch et al. 2012).

The allografts in this study were mixed with gentamicin sulphate, gentamicin palmitate, vancomycin, calcium carbonate/calcium sulphate impregnated with gentamicin sulphate, and calcium carbonate/calcium sulphate bone substitute material (Herafill^®^) impregnated with vancomycin. Local administration of antibiotics delivered from cement was introduced in orthopaedic surgeries in 1970 (Buchholz and Engelbrecht 1970). Cancellous bone grafts were reported as antibiotic delivery system and bone grafts are commonly used to augment bone defects (Lindsey et al. 1993; Goldberg 2000). Impacted morselized allograft bone is a recognized method to obtain additional support for arthroplasty in revision surgery (Toms et al. 2004; Oakes and Cabanela 2006; Barckman et al. 2014). Antibiotic-supplemented impacted bone grafts improve outcome in revision surgery of infected endoprostheses since systemic applied antibiotics do not reach sufficient concentrations around the grafts (Buttaro et al. 2005; Winkler et al. 2006; Barckman et al. 2014). Surgical revision of arthroplasty without cement but augmentation with bone grafts improves the bone stock and might be beneficial for the longevity of the implant and further revision surgery since the number of cementless primary joint replacements is increasing in many countries. Antibiotic-loading of bone grafts seems appropriate to deliver adequate local concentrations similar to PMMA and even higher initial release within 24 h. Good restoration of bone stock and low infection rate after revision of total hip replacements was shown for vancomycin-loaded impacted bone allograft (Buttaro et al. 2005; Winkler et al. 2006) since in vitro studies have shown the ability of bone grafts to deliver antibiotics (Witso et al. 2005; Coraça-Huber et al. 2013a, b; Barckman et al. 2014; Coraça-Huber et al. 2015).

The mixing of bone allografts with antibiotic salts in this study was carried out manually. As this procedure can be easily applied, we affirm that this is a suitable method for an operation room. As we used as one of the reference tests, some authors first dilute the antibiotic powder in a saline solution and then soak the bone grafts in this solution before use (Winkler et al. 2000; Witso et al. 2005). We believe that this is an efficient method for bone chips incorporation with antibiotics since the tissue would act as a sponge absorbing the solution. According to these authors, that could also be an alternative for long-term storage of the grafts with antibiotic solutions. However, according to Sorger et al., the preservation of the grafts for up to 100 h in an antibiotic solution might influence the mechanical stability of the bone (Sorger et al. 2001). Based on Parrish et al. (1973), mechanical testing of osteochondral and structural allografts impregnated with antibiotics in solutions should be performed before this option is taken into clinical use.

In this study, drug concentrations were determined using a conventional microbiological agar well diffusion assay with *Bacillus subtilis* as indicator strain (Stevens et al. 2005; Witso et al. 2005). Because of the hydrophobic profile of GP which does not allow the obtainment of a homogeneous elution, we suggest this method for the concentration estimation instead of spectrometry techniques which could not show accurate results in these conditions. Due to its hydrophobic profile, it is expected that the GP coats not only the bone tissue but also the fat around the BCh (fresh frozen samples), which could increase the adsorption areas of the carrier. In this study, samples coated with GS + GP showed higher and longer release rates compared to the other substances. This could be due to its hydrophobic profile and affinity with the graft’s fat tissue in some cases. Therefore, it could be an advantage of the combination of two the gentamicin salts (GS + GP), comparing with pure or other hydrophilic drugs that its concentrations are kept at homogeneous and constant rates. This could improve the protection of the bone grafts against infections for longer periods.

For the bacterial susceptibility, we tested the eluted substances against *S. aureus* and methicillin-resistant *Staphylococcus aureus* in this study. A range of bacterial species have been implicated in bone and joint infections, although staphylococcal species have been consistently shown to be the most common causative agents, representing approximately 75 % of all strains. Among the staphylococci, *Staphylococcus aureus* remains a frequently isolated pathogen, causing 30–35 % of all orthopaedic implant related infections (Arciola et al. 2005; Schäfer et al. 2008; Esteban et al. 2010; Montanaro et al. 2011; Schwotzer et al. 2014) especially methicillin-resistant *S aureus* (MRSA) (Parvizi et al. 2009).

PMMA beads showed a constant delivery of gentamicin within 14 days with a peak release at day 2 since lyophilized allogeneic bone revealed a constant decline of delivery both providing an inhibition of *S. aureus* and MRSA after 2 weeks. Herafill^®^ beads did not deliver antibiotics longer than day nine and Herafill^®^ and PMMA had a high peak release at day two compared with lyophilized allogeneic bone. Besides the positive results with PMMA in this study and the fact that PMMA is the gold standard biomaterial for local delivery of antibiotics, the efficacy of this material bears many limitations. Such shortcomings include limited antibiotic release, incompatibility with many antimicrobial agents, and the need for follow-up surgeries to remove the non-biodegradable cement before surgical reconstruction of the lost bone (Inzana et al. 2016). Herafill^®^ on the other hand could be used as adjuvant in the bone impacting surgeries once it offers the mechanical stability and capacity of antibiotic local delivery. The capacity of bone grafts to act as gentamicin carriers once mixed with Herafill^®^ granules has been confirmed by Coraça-Huber et al. The combination of the Herafill^®^ granules in different sizes with two gentamicin salts (GS + GP) showed equivalent efficacy against *S. aureus* and *S. epidermidis* (Coraça-Huber et al. 2015).

The release of gentamicin from lyophilized allogeneic bone was similar to the release rate from fresh frozen bone during all the experimental time. That fact might be related to the similar porosity and microstructure of the bone chips (Witso et al. 2002). The release of gentamicin from lyophilized and fresh frozen bone was high in the first and second delivery day, decreasing and keeping a low rate until the end of the second week. Similar pathway for the delivery of antibiotic from bone samples was observed and described by Buttaro et al. 2005 and Winkler et al. 2008 where high initial release of antibiotics for cancellous bone was detected as well.

The release of vancomycin from lyophilized bone was effective against *S. aureus* until the 9th release day and MRSA only until 7th release day. Better results were observed by the release vancomycin from lyophilized bone mixed with Herafill^®^. Here Herafill^®^mixed with vancomycin showed effect against *S. aureus* until day the 14th release day (8 % VB) and MRSA until the 13th release day (8 % VB). Witso et al. also showed the total elution time for vancomycin of 26–32 days in one of his studies using human cancellous bone as bone carrier (Witso et al. 2002). Witso did not describe a difference of release of vancomycin depending on the degree of morselizing of cancellous bone. Lyophilized bone mixed with Herafill^®^G (up to 6.4 % GB) releases effective rates of antibiotics against *S. aureus* until 8th day and MRSA until 9th day.

In conclusion, lyophilized and fresh frozen bone chips showed a release rate of GB from 10 to 0.3 mg/mL from 1st to 4th day (FF-BChT) and from 4 to 0.4 mg/mL from 1st to 3rd day. Although it is a low concentration of gentamicin, based on the literature this amount would be enough to reach the minimal inhibitory concentration (MIC) required for killing *S. aureus* in planktonic form (Alt et al. 2004; Coraça-Huber et al. 2012a). In this way, during the period of 7 days after implantation, the surgical site would be protected against bacterial infection. Until the end of 14 days, the release was low but still efficient to reduce bacteria counts. To maximize the delivery and protection against infection, higher concentration could be loaded to the bone allografts prior implantation.

## Conclusion

Lyophilized and fresh frozen bone allografts once used as antibiotic carrier, provide efficient release concentration to inhibit bacterial growth in vitro. The protection of the grafts up to two weeks depending on the amount of antibiotic loaded and a combination of hydrophilic and hydrophobic antibiotics (e.g. gentamicin sulfate and gentamicin palmitate) are recommendable. The use of lyophilized and fresh frozen bone allografts as antibiotic carriers is recommended for prophylaxis of bone infection.
